# BREAKING GROUND IN TRANSLATIONAL GEROSCIENCE: FROM BIOMARKERS TO CLINICAL TRIALS

**DOI:** 10.1093/geroni/igac059.649

**Published:** 2022-12-20

**Authors:** Jamie Justice

**Affiliations:** Wake Forest School of Medicine, Winston-Salem, North Carolina, United States

## Abstract

The geroscience hypothesis posits that common biological mechanisms of aging drive susceptibility of aged individuals to functional decline, multi-morbidity, and death. The promise of geroscience is that some of these mechanisms may be intervenable, thereby preventing or delaying declines, and providing new therapeutic opportunities for hard-to-treat chronic diseases. This is supported by specific examples of translational research models, and interventions that are at the point of entering human clinical trials. This award presentation will review how we are reimagining existing resources and creating new translational frameworks to test the geroscience hypothesis in humans.

